# Low-grade periprosthetic knee infection: diagnosis and management

**DOI:** 10.1007/s10195-014-0294-y

**Published:** 2014-05-14

**Authors:** Michele Vasso, Alfredo Schiavone Panni

**Affiliations:** Department of Medicine and Science for Health, University of Molise, Via Francesco De Sanctis, Campobasso, Italy

**Keywords:** Total knee arthroplasty, Low-grade infection, Diagnosis, Debridement, Reimplantation

## Abstract

Diagnosis and management of low-grade periprosthetic knee infection are still controversial and debatable. The diagnosis of low-grade infection after total knee arthroplasty is often complex, as clinical symptomatology and diagnostic studies are highly conflicting and knees often exhibit well-fixed components. Although the criterion standard for staged reimplantation is interim placement of an antibiotic-loaded spacer, less-invasive surgical procedures have been advocated for managing infections caused by low-virulence bacteria. Debridement with polyethylene exchange and single-stage reimplantation could offer advantages, such as fewer surgeries, reduced potential for intraoperative complications, and lower direct social costs. The aim of this narrative review was to analyze the literature to evaluate the effectiveness of different surgical procedures in managing low-grade periprosthetic knee infections. Additionally, the most reliable investigations for diagnosing total knee infection caused by low-virulence bacteria were reviewed.

*Level of evidence* Level V.

## Introduction

Infection continues to be a rare but devastating complication of total knee arthroplasty (TKA), occurring in 1–3 % of cases [1–5]. Despite the low incidence, infection is associated with patient morbidity, increased healthcare costs, and recurrence and is also challenging to control [6]. TKA infections are often attributable to staphylococci and streptococci, whereas aerobic gram-negative bacteria cause 10–20 % of all infections and anaerobic bacteria are responsible for another 10 % [7]. Methicillin-sensitive staphylococci, streptococci, and anaerobic cocci are commonly considered as low-virulence bacteria causing low-grade periprosthetic infections; methicillin-resistant staphylococci, enterococci, and gram-negative organisms are certainly considered bacteria of high virulence due to their intrinsic resistance to antimicrobial agents and antibiotics [8]. The diagnosis of a TKA infection, itself very challenging, becomes highly complex in the presence of low-virulence organisms, as clinical features and diagnostic tests may be conflicting. Common clinical signs of infection are often absent, and a gold standard for preoperative diagnosis does not exist.

Whereas two-stage reimplantation is considered worldwide to be the most successful procedure in treating TKA infections, regardless of the etiology of the infecting organism(s) (and timing of infection) [2, 4, 6, 9–12], treatment of a low-grade prosthetic knee infection remains controversial and debatable. Less-invasive and more viable surgical procedures have been advocated for managing low-grade TKA infections (especially if early): debridement with insert exchange and single-stage reimplantation has the advantage of less surgery, ability to maintain motion and soft-tissue health, and lower costs. Furthermore, these procedures mean that the patient is never without prosthetic components, clearly improving comfort [1, 12–14]. Regardless of the virulence of the infecting organisms, debridement and component retention results in inconsistent infection control rates of 16–80 % [15], whereas single-stage revision, in which new, sterile components are implanted and secured with antibiotic-loaded bone cement, presents a large variability in successful infection control of 73–100 % [6, 15]. However, few data exist about the success rates of these different treatments strategies in TKA infections caused by low-virulence bacteria. Therefore, the purpose of this review was to analyze the literature to determine the most effective surgical treatment for managing low-grade periprosthetic knee infection. We also reviewed the most reliable investigations for diagnosing TKA infection caused by low-virulence bacteria.

## Diagnosing low-grade periprosthetic knee infection

Diagnosing low-grade infection after TKA is often highly complex, as clinical symptomatology and diagnostic studies may be conflicting. Moreover, patients often present with well-fixed components, even in in acute infections. We believe the diagnostic process should be developed using the following steps.

### History and clinical features

Early postoperative low-grade TKA infections generally present only with moderate knee pain and stiffness and/or difficult and delayed rehabilitation; persistent fever, severe pain, local warmth, erythema, and swelling are often absent. Hematogenous low-grade infection is quite rare and characterized by a sudden and unexpected deterioration in a previously well-functioning joint. There may also be a history of acute illness followed by sudden deterioration in knee function, with fever and chills [14, 16]. Chronic low-grade TKA infections do not show evident onset. Classic presentation with pain, fever, and local signs such as sinus tract, redness, and swelling is uncommon. More frequently, patients claim only moderate pain and stiffness, which exist since knee replacement [14, 16].

### Radiographs

Standard X-rays are not very useful in the diagnosis of low-grade infection after TKA, particularly in acute infections in which X-rays are always normal. Radiographs showing periosteal new bone formation, scattered foci of osteolysis, and subchondral bone resorption are highly suggestive of infection but typically may be late findings. Periprosthetic radiolucency may be unrelated to a septic process, and serial radiographs help rule out other conditions, such as wear, osteolysis, or fracture [16].

### Laboratory findings

Peripheral white blood cell (WBC) count is frequently normal in low-grade TKA infections and affords little diagnostic help [17]. Erythrocyte sedimentation rate (ESR) and C-reactive protein (CRP) remain the most useful laboratory investigation, even though their sensitivity may be diminished [19]. An ESR >30 mm/h or CRP >10 mg/L should be considered abnormal [7, 18]. Due to this lower sensitivity, serum interleukin-6 (IL-6) could represent a more reliable marker of periprosthetic low-grade infection [17, 19]. With a threshold of <10 pg/ml, serum IL-6 test shows sensitivity, specificity, positive predictive value (PPV), negative predictive value (NPV), and accuracy of 1.0, 0.95, 0.89, 1.0, and 97 %, respectively, thus being a more accurate marker than ESR and CRP [17, 19]. Due to its excellent sensitivity, IL-6 could be an optimal tool for diagnosing low-grade TKA infection [16, 19].

### Radionucleotide scanning

Technetium-99 bisphosphonate scan in conjunction with indium-111-labeled leukocyte scan can contribute to the diagnosis of low-grade TKA infection. Technetium scan is quite reliable in detecting bone-remodelling changes around prosthetic components; however, when positive, it cannot distinguish between aseptic loosening and infection [22]. It has a low NPV in low-grade infections: a normal scan suggests that loosening is not the likely cause of pain, but it does not rule out the possibility of infection [16]. Leukocyte scan is more sensitive but has low specificity. Combining a leukocyte with a technetium scan improves the accuracy for detecting low-grade infection [16]. In particular, if uptake on leukocyte scan is more intense than on technetium scan, it is probable that the TKA is infected. Isotope scanning may present false-positive results: within the first postoperative year, increased scan activity may be present around 85–90 % of tibial and 60–65 % of femoral components in asymptomatic knees [23].

### Aspiration

In the setting of a low-grade infection, knee aspiration could present poor sensitivity due to the low bacterial load. Antibiotic therapy must be suspended at least 2 weeks before aspiration to avoid further false-negative results [18]. The aspirate should be sent for aerobic, anaerobic, and fungal cultures. If the first aspirate is negative, then at least two additional aspirations should be performed [23]. Specimens obtained from the joint must be separated into two or three samples: if all samples are positive for the same organism, the aspiration is considered positive; if only one sample is positive or presents an unexpected positive result, aspiration must be repeated given the high suspicion of contamination [24]. Synovial-fluid WBC count and differential are two helpful parameters: cutoff values for optimal accuracy iare >1,100 cells/mm^3^ for fluid leukocyte count and >64 % for neutrophil differential [25].

### Molecular tests

The detection of bacterial DNA or RNA in synovial fluid using polymerase chain reaction (PCR) studies could be helpful in low-grade TKA infections in which bacterial load is low. PCR amplifies strains of bacterial (deoxy) RNA and can detect nonviable bacteria that do not grow on culture as well as bacteria lysed by ultrasonication, providing results within 12–13 h. Results of PCR are unaffected by the administration of antibiotics [16]. However, molecular techniques cannot distinguish between live or dead organisms, generating false-positive results (low specificity) and therefore being of little clinical utility [26].

### Intraoperative histopathology

Gram staining of intraoperative specimens obtained from the joint capsule or periprosthetic membrane has been reported as being of little help in diagnosing low-grade TKA infection [27]. Intraoperative frozen section for identifying neutrophils in periprosthetic tissue is used to help intraoperative decision making. The exact histologic criteria used for diagnosing infection are not uniform. However, five to ten polymorphonuclear leukocytes per high power field (×400) (PMNs/HPF) in at least five fields is considered consistent with infection [7, 16]. This method is highly dependent on the tissue selected and interpretation by the pathologist. The poor sensitivity of this technique in low-grade infection is probably due to the low inflammatory response caused by coagulase-negative staphylococci, the organisms most commonly infecting TKA [7].

### Sonication

At the time of prosthesis removal, sonication could improve sensitivity of microbiological investigations that, as mentioned above, is significantly diminished in low-grade infections [28]. Sonication uses ultrasound (US) energy to mechanically disrupt biofilm on removed implants following revision surgery. This increases the number of bacteria isolated on culture, or molecular techniques enabling detection of bacteria that would have been missed by conventional tissue culture [28]. Improvement in sensitivity is particularly notable in TKA infected by low-virulence organisms and in patients on antibiotics within 2 weeks prior to surgery [21].

## Surgical management of low-grade periprosthetic knee infection

The goal of treatment is infection eradication and maintenance of a pain-free and functional joint. Different treatment options have been advocated: suppressive antibiotic therapy, irrigation and debridement, single-stage reimplantation, two-stage reimplantation, salvage procedures, and above-knee amputation. Whereas two-stage exchange is considered the best approach regardless of the infecting organism virulence and timing of infection [2, 4, 6, 9–12], optimal management of low-grade TKA infection remains controversial, as open debridement with insert exchange and single-stage reimplantation have been proposed as valid surgical alternatives with the least impact. Suppressive antibiotic therapy has been reserved for patients medically unable to undergo surgery, with limited success rates in low-grade infection [11, 16]. Salvage procedures, such as resection arthroplasty or arthrodesis, and amputation have never been necessary in TKA infected by sensitive bacteria [14]. Therefore, open debridement and single- and two-stage reimplantation are the only procedures reviewed in this paper.

### Irrigation and debridement

In the setting of a low-grade TKA infection, debridement with component retention, and local and systemic antibiotic application could be indicated in healthy patients affected by acute (early postoperative and hematogenous) gram-positive infection with a stable and well-functioning prosthesis and good soft tissue envelope with no fistula [8, 28]. When attempting component retention, thorough debridement and rapid antibiotic treatment prior to the accumulation of biofilm are paramount for a successful outcome [16, 29, 30]. Contraindications are chronic infection, implant loosening, poor soft tissue envelope, and patients with other arthroplasties or a defective heart valve [16, 28]. Polyethylene exchange is always preferred, as it allows complete synovectomy and better debridement of the posterior synovium, and eliminates biofilm formation on the polyethylene [29, 31]. One reason for the failure of arthroscopic debridement is likely due to the inability to eliminate biofilm at the polyethylene–prosthesis interface and debride the posterior aspect of the knee [28, 29]. Intraoperative cultures are performed on synovial fluid and membrane, infected tissues, and polyethylene–implant interface. Organism-specific intravenous antibiotic application is initiated for 4–6 weeks, followed by protracted oral antibiotic administration.

### Single-stage reimplantation

Low-grade infection after TKA has been considered to be potentially susceptible to a single-stage revision due to the low virulence of the infecting bacteria [5, 32, 33]. Factors associated with successful single-stage reimplantation include pathogen identification before revision, infections caused by gram-positive bacteria, absence of sinus tract, and use of antibiotic-loaded bone cement for new component fixation [34]. Single-stage reimplantation involves explantation of all components and cement, thorough debridement, copious irrigation, and reimplantation of new and appropriate prosthetic components with antibiotic-impregnated cement, followed by 6–12 weeks of systemic antibiotic therapy. Then, oral antibiotic therapy should be considered for 3–6 additional months based on recommendations [32]. In low-grade infection, single-stage revision may be advantageous, decreasing recovery time and costs and avoiding some of the problems of two-stage procedures, such as stiffness and arthrofibrosis resulting from a period with a spacer in situ. Furthermore, debridement and a single-stage strategy allows the patient to retain their prosthetic components [13].

### Two-stage reimplantation

Two-stage reimplantation is actually considered state of the art for treating both acute and chronic TKA infections, with reported success rates of 88–96 % regardless the etiology of the infecting organism [2, 4, 6, 9–12]. During the first stage, removal of all components and cement, complete synovectomy, and debridement of all necrotic and infected tissue are performed. Multiple specimens are obtained from deep synovial biopsies and sent for aerobic, anaerobic, and fungal cultures. Resected bone ends and joint space are thoroughly irrigated with pulsatile lavage. Successively, an antibiotic-impregnated cement spacer is positioned into the joint [6, 12, 35, 36]. In low-grade infection, 2 g of vancomycin for a 40-g pack of bone cement could be sufficient [18, 37], as the majority of preformed spacers contain gentamicin. The choice of antibiotic should depend upon the antibiogram: in fact, even though virulence is low, antibiotic susceptibility might be low also [38]. Between stages, targeted intravenously and orally administered antibiotics are generally used for 6–12 weeks on the basis of recommendations of an infectious disease consultant [1, 10].

Decision for second-stage reimplantation is made after a minimum 2-week antibiotic-free interval, when clinical signs of infection have subsided and ESR and CRP levels have steadily trended toward to normal [18, 37]. In low-grade infection, laboratory markers may also normalize in patients with persistent infection, so that knee aspiration before reimplantation should be performed in patients with suspected persistent infection [18, 20, 39]. The second stage includes explantation of the cement spacer, removal of all cement fragments, thorough debridement of the joint and intramedullary canals, copious irrigation, and placement of the appropriate new prosthetic components fixed with vancomycin-impregnated bone cement [18, 40, 41]. After reimplantation, patients receive antibiotics intravenously until intraoperative cultures return to normal.

## Results

In the setting of low-grade TKA infections, irrigation and debridement has shown unsatisfactory success rates, ranging on average from 16 % to 70 % [8, 14]. In acute (early postoperative or hematogenous) infections, primary open debridement is reported as successful in 56 % of patients infected by low-virulence organisms, such as*Staphylococcus epidermidis* or*Streptococcal* species, but only in 8 % of patients infected by *S. aureus* [42]. Assessing 247 knees, Buller et al. [31] reported an overall 45 % failure rate of debridement; higher failure rates were found in infections by resistant organisms and in patients with symptoms ≥21 days. A 34 % failure rate was also reported in low-grade infections. Barberan et al. [43] reported an overall debridement failure rate of 35 % in low-grade infections, which ranged from 17 % in patients with symptom duration <1 month to 69 % in patients with symptoms duration >6 weeks. Better results were reported when considering only early postoperative (and not hematogenous) infections: Kim et al. [44] reported that 27 of 32 knees (84 %) were treated successfully with perioperative debridement.

Certainly, debridement is reportedly unsuccessful for treating chronic low-grade infections, with a final failure rate of 100 % [8, 29, 45]. The most compelling evidence to discourage the use of debridement in low-grade infections is the 34 % failure rate of two-stage reimplantation after failed irrigation and debridement [8]. Fehring et al. [8] suggested limiting its use to early postoperative infections, in which the date of inoculation is well defined and perioperative debridement should improve infection control because intervention may occur before the establishment of drug-resistant biofilm on the implant or before osteomyelitis becomes entrenched in periprosthetic bone.

Reports related to single-stage revision are generally sparse and of poor quality [5]. Single-stage reimplantation has been successful in highly selected cases or small series, with an average success rate of 81 % [5, 12, 15]. In low-grade infections, reinfection rates are reported ranging from 5 % to 11, although in studies with small case series and short follow-ups [5, 11, 13, 34]. Silva et al. [34] reported a success rate of 89 % and found that factors associated with success are absence of sinus formation, gram-positive infection, use of antibiotic-impregnated bone cement in reimplantation, and 12 weeks of antibiotic therapy. Baker et al. [13] reported no significant differences between single- and two-stage revision in terms of postoperative knee score, general health perception, or satisfaction in patients with low-grade TKA infection. They concluded that single-stage treatment may be functionally superior in cases in which infection is successfully eradicated but be may be prone to higher rates of reinfection, which are associated with poorer outcomes. Singer et al. [5] reported that single-stage revision achieved a control rate of 95 % in low-grade TKA infections in which patients with methicillin-resistant*S. aureus* (MRSA), methicillin-resistant*S. epidermidis* (MRSE), or unknown microorganisms were excluded from the study. Higher rates of recurrent infection appeared to be associated with long-term chronic infections of hinged prostheses. Given the very limited number of reliable studies assessing the efficacy of single-stage reimplantation in eradicating low-grade infection following TKA, further investigations are warranted [12].

The success rates of two-stage reimplantation in eradicating infection and restoring function are almost constantly reported to be >90 %, regardless of the etiology of infecting organisms and the timing of infection [2, 4, 6, 9–12], so that this procedure is now considered the most reliable treatment option for infected TKA. Additionally, two-stage reimplantation is reported to be even more successful when used in low-grade TKA infections [2, 4, 9, 39, 46–48]. Volin et al. [48] reported a 94.6 % success rate in methicillin-sensitive *S. aureus* infections. Salgado et al. [47] reported a 17 % failure rate for treated MRSA infections, whereas no failures were noted among infections caused by methicillin-sensitive *S. aureus*. Cordero-Ampuero et al. [9] reported that infection was eradicated in 22 of 25 (88 %) patients infected by methicillin-resistant staphylococci and in 14 of 14 (100 %) infected by methicillin-sensitive staphylococci.

## Conclusions

The diagnosis of a TKA infection, which in itself is highly challenging, may be extremely complex in the presence of low-virulence organisms. Clinical features are often conflicting, whereas classic presentation of pain, fever, and local signs such as sinus tract, redness, and swelling is uncommon. ESR and CRP levels remain the first-line investigation, even though the sensitivity of their results is often diminished in low-grade infections. In these cases, IL-6 levels should support ESR and CRP evaluation to significantly increase the sensitivity of the laboratory findings. Technetium and leukocyte scanning should both be used, and prosthesis sonication may help detect bacteria missed by conventional tissue culture. In patients with an acute postoperative low-grade infection, the strategy of irrigation and debridement with insert exchange persists given the emotional investment in dealing with this complication by both patient and surgeon. Therefore, an attempt to “save the implant” through open debridement appears well intentioned despite a high reported failure rate. In the event of failed debridement or of chronic infections, resection of all components is necessitated. A single-stage exchange has the potential to decrease the number of surgeries and, subsequently, costs. However, infection eradication rates of direct exchange show this method is less safe and predictable than the two-stage revision. This suggests that two-stage reimplantation, with placement of an intrastage antibiotic-loaded spacer, should represent the gold standard for managing low-grade infections.
